# Ophthalmology on Call: Evaluating the Volume, Urgency, and Type of Pages Received at a Tertiary Care Center

**DOI:** 10.7759/cureus.23824

**Published:** 2022-04-04

**Authors:** Heather M McDonald, Yiannis Iordanous

**Affiliations:** 1 Ophthalmology, Western University, London, CAN

**Keywords:** postgraduate education, patient education, resident workload, pager, patient safety, workflow, ophthalmology, on-call

## Abstract

Background: A significant proportion of on-call resident workload is related to answering and managing pages. Ophthalmology residents see high volumes of patients on call, but little is known about the profile of pages they receive. The objective of this study is to characterize the volume, type, and urgency of pages received by the ophthalmology on-call service.

Methods: A retrospective review of on-call pager log sheets and patient charts was performed at a single academic institution. Data were collected from July to December 2019, sampling the first seven days of each month. Data collected for each page included date/time of day, source, and primary concern. For each page leading to a patient encounter, time from page to patient assessment, patient demographics, and final diagnosis were recorded. Continuous variables were reported as mean values, whereas categorical variables were presented as percentages. A two-sample t-test and single-factor analysis of variance were employed.

Results: Over 42 days, 1108 pages were received. Over half of these calls required patient assessment, 71% of which were seen the same day. On average, 26 pages were received in 24 hours. Daytime weekday hours were significantly more busy than weekday nights or weekends (p<0.001). Patients and the emergency department each accounted for almost one-third of calls received. Retina- and cornea-related consults were most common.

Conclusions: Pager volumes in ophthalmology are high and on-call patient volumes are rising. Answering pages increases the on-call resident’s workload and has a negative impact on clinic flow. These data can be used to inform resident curriculum development, hospital system changes, patient education regarding appropriate paging, and medical school teaching.

## Introduction

A significant degree of work for residents on call comes from answering and managing pages. On-call services in ophthalmology receive high volumes of consults, often in the form of pages requiring urgent patient assessment [1,2]. Relative to other surgical specialties, the profile of commonly received pages in ophthalmology may be different, but has never previously been studied. Ophthalmology patients rarely get admitted to the hospital, and therefore, pages from the ward regarding inpatient management are seldom encountered. Instead, the majority of pages are more likely new outpatient consults. Prudhomme et al. found that the ophthalmology on-call service at an academic hospital in Canada received more than double the average number of urgent outpatient referrals from the emergency department (ED) compared to other services, and the second-highest volume overall [1]. They also found that ophthalmology sees these referrals faster than any other service, has the lowest rate of return-to-ED visits, and has the fewest patients lost to follow-up. The referral base in ophthalmology is broad and includes the local ED, optometrists (ODs), general practitioners (GPs), inpatient services, and peripheral general ophthalmologists. Many ophthalmology on-call services also accept calls directly from patients of the local ophthalmology department.

Ophthalmology teaching in medical school curriculums is variable and often limited. Due to inadequate exposure as medical students, the ophthalmological examination, interpretation of findings, and management represent a challenge for many medical professionals outside of the specialty [3]. For this reason, consults from other departments can be vague and inaccurate or lack important details that help in triaging [3]. The concern with incorrect or imprecise referrals is that potentially vision-threatening diagnoses can be missed, which poses a threat to patient safety. In fact, one ophthalmology study found that 36% of OD referrals, 28% of ED referrals, and 32% of GP referrals had an incorrect referral diagnosis [2]. Therefore, if there is any uncertainty from the referring physician or any indication of urgency, ophthalmology services may err on the side of caution and assess patients in a short interval. The wide referral base in ophthalmology and uncertainty of referring healthcare practitioners likely contribute to high on-call consult volumes, which are often received as pages.

To improve on-call workload, clinic flow, and stress for residents, a better appreciation of ophthalmology on-call pages is required. The purpose of this study is to develop a thorough understanding of the type, volume, referral base, and urgency of pages commonly received on call in the ophthalmology department at a single Canadian tertiary care academic center. This study was previously presented as a poster at the 2021 American Academy of Ophthalmology Annual Meeting on November 12, 2021.

## Materials and methods

This study was conducted at an academic ophthalmology department in Canada. Approval was obtained from the Western University Research Ethics Board (REB#115798). The study adhered to the tenets of the Declaration of Helsinki.

All pages received by the on-call ophthalmology resident at this institution are recorded on a standardized call log sheet while the call is being answered. This allows for documentation of referrals and facilitates the transition of care between residents. Referrals through the pager system are the only avenue through which urgent ophthalmology consults are received in this department. This study was conducted as a retrospective review of resident pager log sheets, combined with a retrospective chart review of patients who were urgently referred and seen. The review was conducted from July to December 2019. A sampling technique was employed in which data were collected from the first seven days of each month. This sampling method was derived from a study by Prudomme et al. looking at volumes of outpatient referrals in an ED [1]. This technique was selected in order to account for potential daily, monthly, or seasonal variation in pager volumes. Days were skipped if pager log sheet data were incomplete or missing for that 24-hour period. The seven days had to include one weekend (Saturday and Sunday) and four weekdays (Monday-Friday). The six months of the academic year from January to June 2020 were not included given the onset of the COVID-19 pandemic and presumed alteration to the normal profile of on-call pages during this time.

Log sheets are standardized to include the date, person on call, and call shift. Each documented page has a recorded caller number, caller name, patient name, and information box regarding the call. Data collected for each page included an identifier for the patient, source of the page (e.g. ED, GP, OD, inpatient service, community ophthalmologist, self-referral from patients, other), patient’s presenting complaint, whether the page was a question or a consult, if it was accepted or refused, and approximate time of day the page was received (weekday between 0700h and 1644h, weekday night between 1645h and 0659h, and weekend 24-h period). For each page that was accepted, the patient’s chart was reviewed. Information collected from each patient’s chart included age, gender, time from consultation to examination (same day, next day, later), the type of consult (anatomical location, postoperative, trauma, etc.), and final diagnosis. Chart review also allowed for corroboration of referral source as notes were always dictated back to the referring practitioner. 

The data were analyzed using Excel Statistics. Continuous variables were reported as mean values with standard deviations, whereas categorical variables were presented as percentages. The analysis examined the volume of pages, and potential variation depending on the time of day, day of the week, or month. A two-tailed, two-sample t-test was employed to determine statistical significance between the volume of pages on weekdays versus weekend days and daytime versus nighttime hours. A single-factor analysis of variance was used to determine statistically significant differences in the volume of pages between days of the week and between months. The most commonly encountered final diagnoses were identified and further analyzed to determine how soon pages pertaining to these diagnoses are generally seen. Certain diagnoses, such as retinal detachments, could be further subdivided into different degrees of urgency (i.e. macula-on versus macula-off detachments). Pages received directly from patients were also analyzed as a separate group to determine how many of these pages warranted assessment by the on-call service.

## Results

Forty-two days from July to December 2019 were reviewed in the study, including 30 weekdays and 12 weekend days. A total of 1108 pages were recorded on those days. Fifty-eight percent of pages led to a patient encounter, and there were 780 patient encounters in the urgent clinic during the 42 days included in the study. Some of these patients were walk-ins (i.e. did not page the resident on call before showing up) or follow-up appointments. On average, there were 18.6 patient encounters per day consisting of between 15 and 16 new consults per day, and about three follow-up assessments. Demographics of patients assessed demonstrated an equal number of male and female patients (49.8% males). The average patient age was 53.6 years, with the youngest being three days old, and the oldest being 96 years old.

Table 1 demonstrates the volume of pages received during different time intervals. There was no statistically significant difference in the volume of pages received based on the day of the week (Monday to Friday) (p=0.27) or month (July to December) (p=0.92). The volume of pages on weekdays was significantly higher than on weekend days (p<0.001), as was the volume of pages during daytime hours compared to nighttime hours on weekdays (p<0.001).

**Table 1:** Average number of pages received based on interval of time. SD: standard deviation.

Interval	Average Number of Pages	SD (±)	Range
24-h Period	26	9	6-41
Weekday 24h	31	5	19-41
Weekday Daytime (0700-1644h)	25	5	14-34
Weekday Nighttime (1645-0659h)	6	2	2-13
Weekend 24h (Sat/Sun)	14	4	6-21

**Figure 1:**
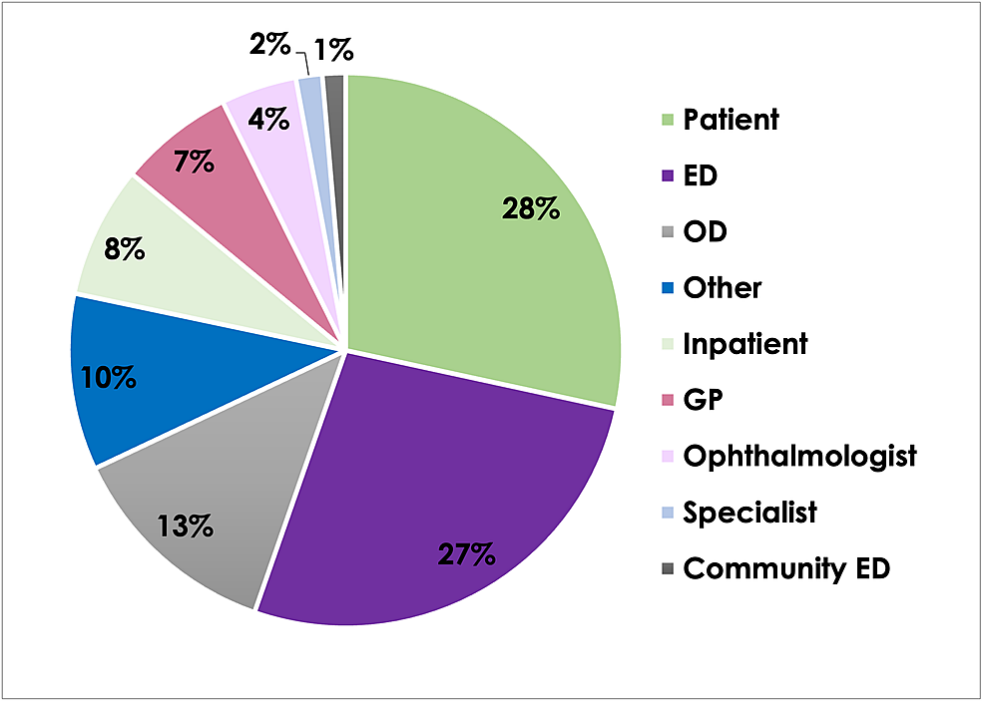
Percent of pages received from each different source. "Other" pages included pharmacy, nursing, microbiology/lab, admitting, and unknown sources. ED: emergency department; OD: optometrist; GP: general practitioner.

The majority of pages received were either from patients directly or from the local ED (Figure 1). Pages from ODs, inpatient teams, GPs, peripheral ophthalmologists, outpatient specialty clinics, and peripheral EDs were also common (Figure 1). Pages from the pharmacy, nursing, microbiology/lab, the admitting desk, and unknown sources were grouped and accounted for 10% of pages. Only 18 pages (1.6% of all pages) were from an unknown source that could not be determined due to inadequate documentation. Of the 1108 pages received, 635 were new consults. Five hundred and thirty-one (84%) of the new consults were accepted and offered appointments. Three-hundred and fourteen (28%) of the 1108 pages were directly from patients, and 155 (49%) of these calls were deemed by the resident on call to warrant assessment. The remainder of patient calls either required reassurance from the on-call resident or pertained to questions regarding their care, scheduling, or prescriptions.

The vast majority of pages from the ED, ODs, GPs, ophthalmologists, and outpatient specialty clinics were new consults. There were differences in the volume of pages and time to assessment depending on the source of the page (Table 2). In total, 71% of pages that were accepted were seen the same day, 25% were seen the next day, and the remainder was seen beyond this point. Certain diagnoses were seen more urgently than others (Table 3).

**Table 2:** Source-based analysis of pages. Volume of pages received, pages that were new consults, new consults that were accepted and seen, and interval of time in which the consult was performed. ^a^Included neurology, nephrology, plastic surgery, hematology, otolaryngology. ^b^The remainder of pages were from other sources including community ED, pharmacy, nursing, microbiology/lab, admitting, and unknown sources. ^c^In the context of patient pages, “consult” refers to all patient pages as none were true consults. ^d^The remainder of consults were seen ≥2 days after the page was received. ED: emergency department; GP: general practitioner.

Source of Page	Number of Pages^b^	New Consults (% of Pages From Source)	Consults Accepted (% of New Consults)	Consults Seen (% of New Consults)	Same Day (% of Consults Seen)	Next Day^d^ (% of Consults Seen)
Patient	314	0 (0)	155 (49)^c^	129 (41)^ c^	99 (77)	29 (22)
ED	298	288 (97)	264 (92)	258 (90)	203 (79)	53 (21)
Optometrist	140	137 (98)	113 (82)	112 (82)	74 (66)	33 (29)
Inpatient	85	50 (59)	34 (68)	30 (60)	13 (43)	9 (30)
GP	74	74 (100)	63 (85)	60 (81)	41 (68)	19 (32)
Ophthalmologist	49	49 (100)	44 (90)	43 (88)	22 (51)	17 (40)
Specialist^a^	17	15 (88)	12 (80)	11 (73)	7 (64)	4 (36)

**Table 3:** Diagnoses more commonly assessed on the same day as the page was received. RCES: recurrent corneal erosion syndrome; IOP: intraocular pressure; NAION: non-arteritic ischemic optic neuropathy; GCA: giant cell arteritis.

Diagnosis (Total Seen)	Percent Same Day
RCES (7)	100%
Optic neuritis (6)	
Corneal ulcer (5)	
Exogenous endophthalmitis (2)	
Globe rupture (1)	
Iritis/uveitis (34)	85-90%
Corneal abrasion (30)	
Keratitis (26)	
Conjunctivitis (24)	75-84%
IOP problems (22)	
Optic neuropathy (NAION or GCA) (14)	

Some on-call diagnoses were very common, presenting on average in at least one patient per day (Table 4). Retinal tears/holes/lattice were the most common diagnoses, and retinal detachments were the second most common. Thirty-four percent of detachments were macula-on, 35% macula-off, 11% macula-split, and 19% unspecified. Macula-on detachments were more frequently assessed on the same day (81%), compared to macula-off or -split detachments (50% and 44%, respectively). Forty-four percent of all patient encounters that transpired following a page were related to a retinal pathology, 21% related to corneal pathology, and 10% related to the lids/orbit. The remainder were divided among neuro-ophthalmology, conjunctiva, postoperative concerns, trauma, uveitis, glaucoma/intraocular pressure, lens, normal exams, sclera, and pediatrics (in order of most to least common).

**Table 4:** Most common diagnoses made for patient encounters occurring secondary to on-call pages. IOP: intraocular pressure.

Most Common Diagnoses	Daily (24-h) Average	Weekly (7-day) Average
Retinal tear/hole/lattice	2.50	17.50
Retinal detachment	1.88	13.17
Posterior vitreous detachment	1.64	11.50
Surface disease/blepharitis	1.05	7.33
Normal	0.95	6.67
Uveitis	0.81	5.67
Corneal abrasion	0.71	5.00
Keratitis	0.62	4.33
Conjunctivitis	0.57	4.00
IOP problems	0.52	3.67
Herpes zoster ophthalmicus	0.38	2.67

## Discussion

Based on the results of this study, it is clear that the volume of pages and patients being assessed through the on-call system in our ophthalmology department is high. Compared to a previous study in our department carried out in 2011 [2], the volume of daily patient encounters has increased from 10 in 2011 to 18.6 in 2019. While this is limited to one ophthalmological institution, studies performed in other specialties have demonstrated a similar rise in consult volume across Canada [4]. This increase in patient numbers may be at least partially accounted for by a growing and aging population [5]. However, the number of residents managing this workload has not changed for many programs, and, therefore, the resident workload has substantially increased [6].

On average, ophthalmology residents receive 25 pages from 0700 to 1644h on a weekday in our department (Table 1). This study was unable to assess the duration of each page, but previous studies calculated an average of 80 seconds per page [7], equating to 33 minutes per business day spent answering pages. High volumes of pages and time spent answering pages cause strain on the healthcare system. One study found that patient length of stay in the ED increased from an average of 7 hours to 12 hours when paging conditions were heavy [8]. Previous studies have also shown that disruption to patient care can lead to medical error [9]. Being paged approximately every 23 minutes (an average of 25 pages in 9.75 hours) throughout the day is a substantial source of distraction and interruption to patient care and workflow [10,11]. This can contribute to resident burnout, reported to be between 41% and 61% among ophthalmology residents [12,13]. In one study, ophthalmology residents averaged 3.6 hours of sleep on call, compared to 6.5 hours when not on call [14]. In our study, residents averaged six pages per night (range 2-13), which could potentially be very disruptive to sleep and contribute to burnout.

On-call consult and pager volumes in ophthalmology can be compared to other on-call services in hospitals. The on-call otolaryngology service in Calgary, which has the same number of residents as our program, saw an average of six consults per day in 2015 [4], in comparison to our 15-16 new consults daily. Witherspoon et al. looked at pages received by different services at The Ottawa Hospital [15]. Orthopedic surgery at one campus received an average of 30 pages per day, similar to our 26 pages per 24-hour period. A significant difference in our study is that the majority (86%) of pages were new consults or direct calls from patients, and 58% led to a new patient encounter. Seventy-five percent of calls in the aforementioned study were from inpatient units [15], which often represent ward issues regarding active patients. Previous studies have shown that inpatient on-call services receive higher pager volumes during evening hours [7,15]. Our study showed that ophthalmology pager volumes are significantly lower during evenings/overnight. This likely reflects the referral base for pages in ophthalmology, which often come from daytime clinics and patients.

In the current study, patients and ED calls were the most common sources of pages received (Figure 1). Over 75% of these patients who required assessment were seen the same day (Table 2). Patient self-referrals generally need to be seen quickly as they are relatively undifferentiated. ED referrals are often urgent, but a secondary factor in the patient receiving a same-day assessment is convenience, as the patient is already in the building. The majority (>60%) of OD, GP, and specialist consults were also assessed the same day (Table 2). Although the final diagnosis did not always require urgent specialist consultation (Table 3), vague referrals require a timely assessment to rule out vision-threatening diagnoses [2,16]. A lower percentage of inpatient and community ophthalmology referrals were seen on the same day (43% and 51%, respectively), likely due to travel time from other hospitals and towns, respectively. Only 49% of pages from patients led to an in-person assessment (Table 2), with many pages pertaining to questions, prescription refills, and other non-urgent, miscellaneous concerns. A large proportion of pages received by other on-call services are also non-urgent [7,17], placing unnecessary strain on the system.

Retinal pathology makes up the largest proportion of urgent consults being referred through pages (44%), with cornea being the second most common (21%). Procedures can be time-consuming, and on average there are over two retinal tears/holes/lattices that require laser retinopexy by a resident each day (Table 4). Retinal detachments are also very common, presenting on average almost two times per day. Each patient requires an in-depth conversation about the risks and benefits of surgery and the logistic organization of the operation.

Several lessons can be learned from this data. First off, given the high volume of pages and patient encounters, triaging skills for residents are critical and should be actively taught. Medical students are generally underprepared for clinical decision-making and clinical prioritization, especially when disruptions are present [18]. The MyOnCall Pager app was designed with the purpose of teaching and testing these skills, with preliminary results showing a positive impact on learning [19]. Simulated teaching and testing of pager management should be integrated into postgraduate curricula. Second, paging volumes can serve as a marker for resident workload that may not be captured by looking at duty hours alone, and should be monitored when considering systemic issues like operational strain and resident burnout [8]. There may be a role for a triaging nurse to hold the pager and help resident workflow [20]. Third, patients need to be educated during their hospital visits regarding what the appropriate reasons are to call the on-call resident. Fourth, improvements must be made to medical school teaching of clinical ophthalmology, allowing for more thorough and confident assessment and management by primary care providers. With adequate training, primary care physicians should be able to confidently diagnose and manage straightforward ocular conditions, such as corneal abrasions, ocular surface disease/blepharitis, and conjunctivitis.

Limitations of this study include that it was conducted retrospectively. The data were extracted from resident pager log sheets, which are used as an organizational tool but are not mandatory, making documentation variable. For this reason, the first seven days of data available for each month did not always correspond to consecutive days. It is possible that not all pages were recorded and, therefore, this underrepresents the true volume received. A very small minority of pages (1.6%) could not be further analyzed due to a lack of detailed documentation (unknown source). If a certain type of page is less likely to be documented, this would bias the results. The study methods did not allow for collection of data regarding the duration of each page, the exact time of each page, or the exact length of time from page to patient assessment. Weekends could not be further subdivided into daytime and nighttime as these pages were not distinguished on log sheets. Another limitation is that this was a single-center study. There is regional variability with regard to how academic centers run their emergency eye clinics, including who is holding the pager (i.e. consultants, junior or senior residents, administrative staff), who can page on-call (i.e. direct patient calls), and how urgent referrals are received (i.e. faxes versus pages). While these differences may limit the generalizability of our results, the total number of residency positions in ophthalmology across Canada has not changed since 2012 [5]. Therefore, at least some of the problems identified in this study, such as the burdensome pager workload and increasing on-call patient volumes, likely exist in other academic hospitals across the country. 

## Conclusions

Overall, this study demonstrates a high pager burden for ophthalmology residents trying to balance a busy on-call clinic. These volumes have risen dramatically over an eight-year period, but no system changes have been implemented to account for this. Interventions such as simulated training for managing pages, hiring of a triaging nurse to hold the pager, patient education regarding calling the resident, and improved teaching of ophthalmology in medical school would likely help improve the currently over-burdened system. Larger studies are required to determine if the same problems exist nationally and internationally. However, given growing and aging populations worldwide, some of these interventions may be beneficial to other ophthalmology residency programs managing challenging pager and patient volumes.
